# Facilitators and barriers to monitoring and evaluation at syringe service programs

**DOI:** 10.1186/s12954-024-01073-z

**Published:** 2024-08-28

**Authors:** Elise Healy, Arianna Rubin Means, Kelly Knudtson, Noah Frank, Alexa Juarez, Stephanie Prohaska, Courtney McKnight, Don Des Jarlais, Alice Asher, Sara N. Glick

**Affiliations:** 1 Division of Allergy and Infectious Disease, School of Medicine, University of Washington, 325 9th Ave, Box 359777, Seattle, WA 98195 USA; 2 Department of Global Health, University of Washington, Seattle, Washington USA; 3 Office of Infectious Disease, Washington State Department of Health, Olympia, Washington USA; 4 Dave Purchase Project, Tacoma, Washington USA; 5 School of Global Public Health, Department of Epidemiology, New York University, New York, New York USA; 6 Centers for Disease Control and Prevention (CDC), Atlanta, Georgia, USA

**Keywords:** Syringe service program (SSP), Monitoring & evaluation (M&E), CFIR, Data systems

## Abstract

**Background:**

Syringe services programs (SSPs) provide harm reduction supplies and services to people who use drugs and are often required by funders or partners to collect data from program participants. SSPs can use these data during monitoring and evaluation (M&E) to inform programmatic decision making, however little is known about facilitators and barriers to collecting and using data at SSPs.

**Methods:**

Using the Consolidated Framework for Implementation Research (CFIR), we conducted 12 key informant interviews with SSP staff to describe the overall landscape of data systems at SSPs, understand facilitators and barriers to data collection and use at SSPs, and generate recommendations for best practices for data collection at SSPs. We used 30 CFIR constructs to develop individual interview guides, guide data analysis, and interpret study findings.

**Results:**

Four main themes emerged from our analysis: SSP M&E systems are primarily designed to be responsive to perceived SSP client needs and preferences; SSP staffing capacity influences the likelihood of modifying M&E systems; external funding frequently forces changes to M&E systems; and strong M&E systems are often a necessary precursor for accessing funding.

**Conclusions:**

Our findings highlight that SSPs are not resistant to data collection and M&E, but face substantial barriers to implementation, including lack of funding and disjointed data reporting requirements. There is a need to expand M&E-focused funding opportunities, harmonize quantitative indicators collected across funders, and minimize data collection to essential data points for SSPs.

**Supplementary Information:**

The online version contains supplementary material available at 10.1186/s12954-024-01073-z.

**Table:** 

Text box 1. Contributions to the literature
• SSPs provide lifesaving services but there is a lack of implementation science research to understand and optimize how services are provided.
• Data collection and reporting is an often-mandatory part of SSPs’ operation, but can be a substantial burden on clients and staff.
• We used the CFIR to better understand the facilitators and barriers in SSP M&E systems.
• We found that external funding influences changes to M&E systems and strong M&E systems often facilitate access to funding.
• Our findings highlight a need for more data-specific support at SSPs and for harmonized and minimized data requirements across SSP funders.

## Introduction

Syringe services programs (SSPs) provide harm reduction supplies (such as injection and/or smoking supplies) and services (such as syringe disposal and/or healthcare referrals) to people who use drugs. In other contexts they may be known as needle exchanges, needle-syringe programs (NSPs), syringe access programs (SAPs), among other names. SSPs are effective at preventing HIV and HCV infections, and also provide life-saving overdose prevention education and services, such as naloxone distribution [1–5]. The operationalization of SSPs varies substantially. SSPs can be run by health departments, community-based organizations, federally-qualified health centers, or can be stand-alone grassroots organizations operated by volunteers.

The scope and type of data collected about clients and services provided at SSPs varies depending on the policy environment, SSP priorities, and available resources. The policy environments that govern SSPs vary greatly across the US state by state, and even county by county. At most SSPs, some form of data collection is a requirement, either as a condition of funding or for legal authorization [6]. Common types of data collected include supply distribution, syringes returned, demographic information about clients, and overdose data such as number of naloxone doses distributed and reported overdoses reversed [6]. Less common types of data includes drugs used, method(s) of administration, and personal information such as name and date of birth [6]. Personal identifiers are often not collected because SSPs aim to provide low-barrier services that maintain the confidentiality – or anonymity – of clients, which is driven by the stigma and criminalization of drug use [7].

A recent survey of 63 SSPs found that almost all SSPs surveyed (~ 95%) collected at least some data about their services, and 73% reported using those data for internal purposes [6]. Despite data collection being an almost universal requirement at SSPs, very little literature on data collection experiences and use at SSPs exists. Monitoring and evaluation (M&E) is the process of collecting and analyzing data to determine how well a program is working. M&E can be integral for decision making at SSPs. For example, routine data collection can help inform what services are offered, understand community trends, advocate for interventions to improve the health of people who use drugs, and secure funding [6]. Importantly, a comprehensive understanding of the motivations, facilitators, and barriers of data collection and use at SSPs would provide leverage for implementing improvements in what data are collected at SSPs.

Using the Consolidated Framework for Implementation Research (CFIR), we conducted a qualitative study to describe the overall landscape of data systems at SSPs, understand facilitators and barriers to data collection and use at SSPs, and generate recommendations for best practices for data collection at SSPs. The CFIR is a meta-theoretical framework that provides a menu of multi-level constructs that may influence implementation of an intervention or program [8–10]. The CFIR can be used at any stage of implementation to understand facilitators and barriers of program success and across a wide-range of health innovations such as healthcare delivery, health promotion, and quality improvement [9–18]. Evidence regarding multi-level determinants of data collection and use will be helpful for SSPs in advocating for resources for improved M&E systems. It may also be useful for funders and policy makers to understand the implications of data collection and reporting at SSPs and inform their reporting requirements.

## Methods

From October 2020 to June 2021, we conducted 12 interviews with staff from a sample of SSPs to discuss their organization’s data systems as well as facilitators, barriers, and best practices for implementing M&E activities.

### Study participants

We sampled programs from the national SSP directory maintained by NASEN [19]. This is an opt-in directory of SSPs in the United States, which includes SSP location and contact information. Programs were purposively sampled by NASEN staff based on each SSP’s experiences with M&E. We aimed to interview a minimum of 12 SSPs with a variety of M&E experiences, including those with minimal M&E experience and those with more extensive experience. We reached out to a total of 21 programs via e-mail using the contact information from the NASEN directory before reaching our goal of 12 participants. The job title and role of the interview respondent varied, though all interviewees were familiar with their program’s data system. Interviewees roles ranged from direct service staff to data specialists, to program founders, with many interviewees holding multiple roles at their organizations and some additionally identifying as peers.

### Theoretical framework

This study used the CFIR [8] to inform data collection and data analysis. Specifically, the CFIR was used to develop interview guides, develop an initial codebook, and interpret study findings. For the purposes of this study, we identified 30 of 39 CFIR constructs across all five domains that were most applicable to M&E at SSPs: Intervention Characteristics, Outer Setting, Inner Setting, Characteristics of Individuals, and Process. These constructs were selected as they were most relevant to the research question and a priori hypotheses about factors influencing M&E in SSPs. All constructs and domains have been defined in Table 1.

**Table 1:** Applied CFIR constructs and relevant definitions for assessing monitoring and evaluation (M&E) at syringe services programs (SSPs) in the United States

Domain	Construct	Definition
*Intervention Characteristics*: Aspects of data systems that impact M&E	Adaptability	How an M&E system would need to be tailored to fit the SSP’s environment.
	Complexity	Perceived difficulty of implementing an M&E system into the SSP
	Cost	Cost associated with launching and/or maintaining an M&E system and how that impacts perspectives on M&E
	Design quality & packaging	Perception of the quality of the current M&E system
	Evidence strength & quality	Resources that would support M&E practices, how M&E impacts SSP services, and evidence required to get staff on board for an M&E system
	Innovation source	Who developed the M&E system and what was considered during its development
	Relative advantage	Perceptions of the advantages and disadvantages of changing M&E practices/system
*Outer Setting*: Features of an external legal or social context that may influence implementation of M&E	Cosmopolitanism	The information accessed from other SSPs and if it has any impact on views of M&E systems
	External policy and setting	The performance measures, guidelines, policies, regulations, and/or fiscal/other incentives that influenced the M&E system
	Patient needs and resources	If and how an M&E system would change services provided to clients and if that system would introduce new barriers or facilitators to clients accessing services
	Peer pressure	How an M&E framework could give the SSP an advantage compared to other SSPs
*Inner Setting*: Features of implementing SSPs that may impact implementation or effectiveness of M&E activities (e.g., the organizational goals and priorities of the SSP)	Access to knowledge & information	The type of training the SSP would require to launch and maintain a new M&E system as well as who currently provides M&E related support
	Available resources	Where the majority of the SSP’s funding comes from and if there would be sufficient resources to launch, maintain, and administer an M&E system
	Compatibility	How well a new M&E system would fit within the SSP’s norms and values
	Culture	The SSPs general beliefs and values how those impact implementation of an M&E system
	Implementation climate	The absorptive capacity for change, shared receptivity of involved individuals to an innovation and the extent to which M&E will be rewarded and supported within the SSP
	Learning climate	The extent at which members of the SSP feel like they can try new things to improve work processes
	Organization incentives & rewards	The SSP’s current goals and how they are monitored for progress
	Readiness for implementation	Tangible and immediate indicators of organizational commitment to its decision to implement a M&E system
	Relative priority	The SSP’s current highest priority initiatives
	Structural characteristics	The social architecture, age, maturity, and size of the SSP
	Tension for change	The degree to which the current M&E situation is perceived as needing to change
*Characteristics of Individuals*: Individuals involved in implementation, such as SSP staff and volunteers, and how they engage in data collection and evaluation	Knowledge & beliefs about the intervention	Perception of colleagues’ confidence in implementing a M&E system at the SSP
	Self-efficacy	Individual belief in own capabilities to execute courses of action to achieve M&E goals
*Process*: Strategies and key SSP stakeholders that may influence the effectiveness of M&E	Engaging: Champions	The people within the SSP who will likely champion the M&E efforts
	Engaging: Key stakeholders	The key individuals to get on board with a new M&E system and how they should be engaged
	Engaging: Opinion leaders	Individuals in the SSP who have the most influence over the development/implementation of a new M&E system
	Engaging: Participants	Whether the SSP would need to communicate with clients about changes to the M&E system
	Planning	The degree to which a plan or method for implementing M&E is developed in advance and the quality of those plans

*Abbreviations* M&E, monitoring and evaluation; SSP, syringe services program

### Data collection

A semi-structured interview guide (Appendix I) was developed using all five CFIR domains and 30 relevant CFIR constructs (Table 1). The guide was piloted with a research team member who had experience working at SSPs, and then refined before implementation. Interviews were conducted and recorded over Zoom by two trained and experienced interviewers (NF and EH). In addition to an interviewer, a note taker was present for each interview (EH and KK). At the beginning of each interview, the interviewer obtained verbal consent from the interviewee to participate in the interview and for audio recording. The notetaker documented key SSP characteristics, such as size and location, for each SSP. On average, interviews lasted 2 h and interviewees received a $250 Visa gift card as compensation for their time.

### Data analysis

From July 2021 through March 2022, we prepared transcripts, coded, and analyzed the interviews. A professional transcription service transcribed the interview audio files. The research team randomly spot checked the transcriptions for errors and uploaded finalized transcripts to Dedoose [20] for coding. We developed the codebook using a mixed deductive and inductive approach. An a priori codebook was developed using relevant CFIR constructs as codes and additional codes were inductively added based on the text once coding started to capture details related to the programs’ staffing and data structures. All coders coded two transcripts in full, and two inter-coder reliability (ICR) meetings were conducted to discuss code application and finalize the codebook. Once the codebook was finalized, one coder (EH) primary coded all twelve transcripts. Secondary coders (KK, SNG, and ARM) each secondary coded four transcripts to ensure consistent application of codes. After primary and secondary coding was completed, coders held regular meetings to discuss any disagreements in code application as well as emergent themes and patterns among/between data groups.

After data were analyzed, SSPs were categorized as “high M&E” or “low M&E” capacity. To make these determinations, we considered the program’s data collection norms, data agency, and data utilization. Data collection norms were the degree to which data were regularly collected and how actionable the data were; data agency was the degree to which data collection was driven by external requirements versus an organization’s own internal priorities; and data utilization referred to the extent to which an organization analyzed and used its data—especially for internal purposes. We scored all 12 SSPs as “high”, “moderate”, or “low” for each category (data collection, data agency, and data utilization), and then came to a group consensus about which SSPs were “high M&E” and “low M&E” based on the scoring.

We prepared five memos, one for each CFIR domain, where we summarized the application across all interviews of each relevant construct (as codes) within each domain along with supportive quotes. After the memos were written, we generated heat maps from our memo summaries to visualize and compare the influence of each construct on a program’s M&E capacity based on group type (high vs. low M&E). The analysis team worked together to label constructs based on whether they were a facilitator (blue) or barrier (red) to establishing and maintaining M&E systems at SSPs. We also assessed the strength of their influence and labelled each facilitator and barrier as either high (dark) or low (light). The heat maps generated from the data were used to visually determine which constructs served as common facilitators and barriers of data generation and data use across SSPs, and which constructs differentiated high from low M&E SSPs. The COREQ reporting guideline was used.

### Ethical approval

Due to the programmatic nature of the data collection (i.e., data were not collected about individuals), the University of Washington Human Subjects Division determined that this project was not human subjects research and did not require additional approval. All names and/or identifying information were removed from transcripts to protect the identity of the interview respondents and their associated institutions.

## Results

We conducted 12 individual interviews with staff at SSPs in the United States. Key SSP characteristics are described in Table 2. Six SSPs were categorized as “high M&E” (scored high in data norms, agency, and utilization by research team) and six as “low M&E” (scored lower in data norms, agency, and utilization by research team). There was substantial heterogeneity in M&E systems, including software systems used at SSPs. Most programs used pen and paper for at least some data collection and only one program solely used digital data collection.

**Table 2:** Geographic and organizational characteristics of interviewed syringe services programs (*N* = 12)

City size	*n*
* Metro-urban (> 400*,*000)*	6
* Mid-sized regional cities (80*,*000-400*,*000)*	4
* Small town/rural area (< 30*,*000)*	2
Location	
* Northeast*	3
* South*	3
* Midwest*	3
* Mountain West*	1
* West Coast*	2
Organization type	
* Non-profit*	10
* Health Department*	1
* University*	1
Service provision model*	
* Fixed/Brick and mortar*	8
* Delivery*	4
* Outreach*	4
* Mobile*	6

*Categories are not mutually exclusive

### Facilitators and barriers to M&E at SSPs

**Fig. 1:**
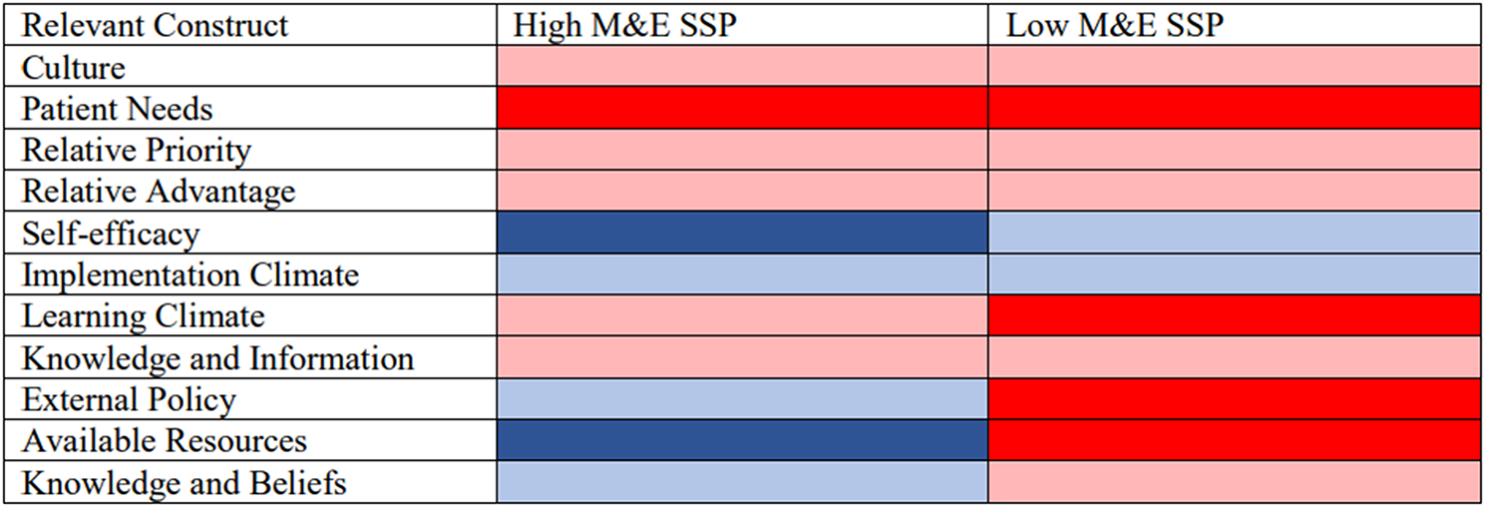
Heatmap of influential CFIR constructs for assessing monitoring and evaluation (M & E) at syringe programs (SSPs) in the United States. Pink: slight barrier, red: strong barrier, light blue: slight facilitator, dark blue: strong facilitator

Four main themes emerged, including that SSP M&E systems are primarily designed to consider perceived SSP client preferences; despite willingness to learn new data systems, SSP staffing capacity influences the likelihood of modifying M&E systems; external funding frequently forces design and changes to M&E systems; and strong M&E systems including data staff are often a necessary precursor for accessing funding, but it often requires funding to build robust M&E systems. CFIR constructs representing facilitators and barriers to M&E practices across SSPs included *Culture*,* Patient Needs*,* Relative Priority*,* Relative Advantage*,* Self-Efficacy*,* Implementation Climate*,* Learning Climate*, and *Knowledge and Information*. The constructs that differentiated high and low M&E SSPs included *External Policy*,* Available Resources*, and *Knowledge and Beliefs* (Fig. 1).

### Concern for client safety and preferences drove M&E design, particularly decisions to minimize M&E activities

Across high and low M&E SSPs, interview respondents reported that providing services to clients was their SSP’s main priority. SSPs were willing to forgo certain data collection activities, e.g., collecting information on income and/or housing status, to prioritize client safety and comfort. When asked about overall organizational goals, most SSPs focused on meeting client needs such as expanding services and/or supplies. For example, some SSP goals included rolling out new HIV-related services or referrals or providing new supplies like smoking kits. Very few SSPs mentioned program goals related to M&E. This theme was driven by the CFIR constructs *Culture*,* Patient Needs*,* Relative Priority*, and *Relative Advantage.*

*Goals and priorities are housing, basic human needs being met… not necessarily attached to treatment, wellness, and recovery… I am of the belief that you have to put the horse before the cart. And if you want people to engage in wellness systems, their needs have to be met.*

*-High M&E SSP*,* mountain west*,* respondent 2*

In many instances, data collection was seen as secondary and even a barrier to service provision. Respondents described how SSP clients are a heavily surveilled and stigmatized population, and expressed concern about collecting data with a population that is criminalized. Respondents across both high and low M&E groups reported that they were concerned for clients’ comfort, safety, and time when collecting data, and acknowledged that sometimes data collection negatively impacted relationship building.

*I think that – data in general is complicated in terms of surveillance and folks that are really struggling with already being surveilled so much. Most people now know [SSP] and can rattle off their client ID, but for new folks, I totally get why you’d be like, why do you need that information or why do you need any of this information?*

*-High M&E SSP*,* northeast*,* respondent 5*

Concerns for client needs shaped data collection activities at both high and low M&E SSPs. Perceptions of client preferences shaped which data are collected and how data are collected. For example, at many SSPs staff reported actively trying to reduce the amount of time data collection takes during their interactions with clients. In many cases this meant providing the option for clients to refuse to answer some or all questions to streamline the interaction.

*We operated for so many years illegally without any authorization, but tolerated, that we’ve just tried to stay on the low end of asking too much of our clients. We just saw it as being intrusive, so we just try not to be.*

*-Low M&E SSP*,* west coast*,* respondent 7*

Some respondents in both high and low M&E SSPs also expressed being unsure of the benefit of supplemental data collection outside of what was requested or required by external entities.

*I don’t like to ask for information… I’d rather ask, how are you doing today, or do you want something to drink? …I don’t like tracking the data, and collecting it, and giving it back because I feel like it’s purely for a purpose that isn’t in the best interest of people that I care about.*

*-Low M&E SSP*,* northeast*,* respondent 3*

Sometimes low M&E SSPs described intentionally collecting less data to ensure their services remained as anonymous and low-barrier as possible. For example, one SSP in a policy restrictive environment intentionally does not collect some of the data required at the locally approved SSP to meet clients’ comfort.

*We have seen some folks come back to us [from the] legal exchanges because the data …[the legal exchange is] collecting [participants] don’t want to give, and so we use that to inform what data we’re willing to collect. Like, we’re not gonna ask folks things that we’ve heard that they don’t wanna provide.*

*-Low M&E SSP*,* south*,* respondent 1*

### SSP staff and volunteers are willing to learn new data systems, but technology and data literacy influences the degree to which an SSP gains and implements new M&E skills

Respondents at both high and low M&E SSPs reported that staff were generally amenable to changes related to data collection. Interview respondents reported that while some staff were attached to current systems, other staff might welcome change. A few programs, in both high and low M&E groups, described staff as being excited about innovations in data systems. In addition, respondents expressed feeling confident that they could learn a new M&E system. This sub-theme was characterized by the CFIR constructs *Implementation Climate* and *Self-Efficacy*.

*…at [SSP], people are very flexible and adaptable… – I don’t think anyone would ever be like, no, thank you [to a new data system]… but when we did launch [current data system], folks were pretty excited, actually, I was surprised, and they were like, oh, I like this, or this makes sense.*

*-High M&E SSP*,* northeast*,* respondent 5*

In general, making infrastructural changes, especially data-related changes, is challenging at SSPs. At both high and low M&E SSPs, technology comfort and data literacy influence the degree to which gaining new M&E skills is feasible. This sub-theme was characterized by the CFIR constructs *Learning Climate* and *Knowledge and Information*.

*It’s really hard to introduce new technologies… it’s not because staff aren’t receptive to it. It’s just that it’s a learning curve for them because a lot of our staff, unfortunately, didn’t have that privilege to have the education learning computers or learning some of these programs… So, we’re providing a lot of that support in having to navigate new electronic systems for a lot of our team members.*

*-High M&E SSP*,* mid-west*,* respondent 6*

Interview respondents at both high and low M&E SSPs reported that there would need to be trainings to make M&E changes at their SSP. Potential trainings would need to span from basic computer skills to tailored trainings on how to use new software.

*I think it’s gonna be a lifelong process of learning, and adapting, and evaluating… I think the biggest challenge is gonna be training the people to use whatever we develop because everybody’s so spread out… And level of ability of using technology – I don’t think everybody knows how to use a tablet.*

*-Low M&E SSP*,* south*,* respondent 11*

In addition, most staff at both high and low M&E SSPs are already stretched thin due to service provision thus the time required to plan and rollout changes is burdensome. Data collection is often just one aspect of a staff members’ role.

*That’s the biggest thing I think about, is how people are already stretched thin.*

*-High M&E SSP*,* northeast*,* respondent 5*

### External entities, including funders and partners, have a strong influence on the type and frequency of data collection at SSPs

At all SSPs interviewed, external entities - such as funders, local governments, and/or partners - influence the type and frequency of data collection and reporting. Despite the overall priorities of SSPs being focused on meeting clients’ needs, SSP data collection activities are typically driven by funders and local or state policies. The CFIR construct influencing this theme was *External Policy.*

SSPs reported constraints on using federal money to purchase syringes, which inhibits programs from being able to fully operate using federal (and often state) grants. As a result, SSPs frequently acquire funding from a mix of private funding, federal and/or state grants, and donations.

*“The majority is private donations and grants. I guess there’s some federal funding as well. There was just a behavioral sciences COVID grant we had. So*,* it’s piecemeal. It comes through an organization here… They’re kind of our parent organization… They buy our supplies*,* and they just give us the supplies to take out.”*

*-Low M&E SSP*,* south*,* respondent 1*

Funders influence which specific variables are collected during M&E. For example, both high and low M&E SSPs reported that some data collected and/or certain data related goals were driven by funders. Types of data often required by funders includes supplies distributed, number of encounters, demographic data, and overdose reversals.

*There’s so many things we wouldn’t collect if it wasn’t for the reporting requirements. And it’s always a balance with our funders of being like, “We can do that,” or, “Hey, maybe that’s crossing the line.”*

*-High M&E SSP*,* northeast*,* respondent 4*

Across both high and low-M&E SSPs, respondents reported that funders rarely provided feedback and/or reports based on the data they submitted.

*Not necessarily [received feedback on reports submitted to the state]. Usually, if I have a question or a concern about some of the data, I know the folks at the state that I can email or even call them up and ask about… if I have a problem I can call them, but they don’t necessarily contact me….*

*-Low M&E SSP*,* west coast*,* respondent 7*

A few SSPs reported collecting data to be able to prove that they are operating in accordance with local policies. Depending on the policy environment, data collection may be integral to ensuring that an SSP can continue to operate. This primarily arose in interviews with organizations that were operating in restrictive policy environments.

*We only collect how many syringes we’ve taken in because if we get arrested, we wanna show, we’re obeying the law. We may not be legal, but we’re at least obeying the spirit of the law. It’s always nice to be able to look at a success story to bring somebody’s unique identifier up and see how they’ve progressed and fulfilled their goals as a [participant], but I don’t think we see that as necessary to us really. Data is – yeah. It’s a requirement to pull for funding, and so that’s we think about it. We hate it.*

-*Low M&E SSP*,* south*,* respondent 1*

High M&E SSPs often had more grants and/or services that required more data collection, whereas low M&E SSPs reported fewer data collection requirements.

*So, like I said, at any point in time, we’re reporting to seven or eight different grants. So, all of them have the things that they ask for, whether it’s quarterly, yearly, or –.*

*-High M&E SSP*,* the south*,* respondent 8*

### There is a bidirectional relationship between M&E capacity and increased funding, where funding may provide an SSP the means to hire data staff, but data staff are instrumental in developing grants to acquire funds

When SSPs receive new funding they often have additional reporting requirements. However, these funds do not always provide the resources necessary to access additional staff who can manage software and analyze data. High M&E SSPs often have hired staff members (or teams) that manage their data system and reporting requirements. However, data collection responsibilities often still fall on outreach workers who are engaging with SSP clients. The CFIR constructs related to this theme were *Available Resources* and *Knowledge and Information*.

*…generally we kind of input into our data management system monthly. So, then I will go into [the data system] …and we enter in all of our numbers monthly approximately and then that’s when it’s handed over to our data team so they can look at all the numbers.*

*-High-M&E SSP*,* west coast*,* respondent 9*

Acquiring funding to support full-time data managers requires personnel who can use the data to tell a compelling story for the funds. Data by itself is not enough to secure funds, but a robust M&E system and personnel who can utilize the data can provide the footing to acquire funding. However, hiring staff to support data requirements is often expensive. One high-M&E SSP reported dealing with this barrier by accessing free grant writing support through a state-wide program for small non-profits that made applying for larger funding opportunities possible.

*what it has morphed into is now an entire team of specialists and technical support folks that go out and help [location]… they had a program this past year that was only for small non-profits and small communities that could not afford grant writing services… they had funds allocated to help us pay for grant writing services… I’ve accessed that system, and that’s what helped me get these two major grants that we - and now I have the ability - now, we’re applying for the [local] funding that I was telling you about earlier. So, that, kind of, elevated all of us to that level of the playing field, that a little more evened out and made us competitive [for grants].*

*-High-M&E SSP*,* mountain west*,* respondent 2*

Low M&E SSPs often lacked the infrastructure to obtain and analyze baseline data, and thus they were not positioned to apply or successfully procure the funding they need to collect, manage, and analyze data. Many low M&E SSPs barely have enough funding to provide services to clients or pay their staff, much less hire a data analyst or grant writer.

*I think just to get some funding to have enough syringes to meet the need… I feel like I beg*,* borrow*,* and steal all the time. I think my estimated supply budget is approximately $100*,*000 a year*,* and I am nowhere near it.*

*-Low M&E SSP*,* mid-west*,* respondent 10*

## Discussion

Due to longstanding barriers to federal funding for SSPs [21], many programs remain under or unstably funded. Moreover, public and political support for SSPs can be volatile between jurisdictions, often requiring SSPs to provide evidence of their program’s success and impact [22]. For these reasons, M&E can be essential for SSPs to evaluate their program operations and impact, while also a hardship for SSPs that lack sufficient staff and resources to do so. To optimize data collection and use without placing undue burden to SSPs, it is important to understand strengths in SSP data collection systems, identify areas for improvement, and make recommendations for more effective data collection and implementation of M&E. We used the CFIR to analyze qualitative data from 12 SSPs about their data systems and experiences to better understand the facilitators and barriers SSPs face in developing their M&E systems.

Despite major software system differences among SSPs, data collection decisions were often driven by staff concerns with client needs and preferences. At all participating SSPs, staff reported that client comfort and access to low-barrier services were more important than data collection. Most interviewees primarily identified data collection as an unwanted burden rather than a potential way to assess how their program’s services were addressing community needs. This finding highlights the tension between data collection priorities and requirements at SSPs, where data collection is often required either through local policies and regulations or through funding requirements [6]. Consistent with several other studies, the CFIR construct *Patient Needs and Resources* had a strong impact on SSP M&E systems. For example, in one study seeking to adapt the CFIR to better fit patient-centered care settings, the *Patient Needs and Resources* construct was considered so crucial as to become its own domain within the framework [23]. Studies from a range of settings have demonstrated that perspectives of care providers on the needs of their patients greatly impacts the implementation of programs, including substance use treatment programs [11, 15, 17, 23]. Our findings align with previous research and indicate that the design of M&E systems is highly influenced by staff concerns for client perspectives.

While participating SSP staff were not resistant to potentially changing M&E systems, the overall culture and priorities of SSPs were not conducive to rigorous data collection. For example, staff expressed willingness to learn new M&E-related skills, but attaining these skills was not often feasible due to limited organizational and staff bandwidth and the need for additional training. The SSPs with prior experience updating M&E systems noted that variability in staff knowledge and comfort with technology required different types of training for different staff. This indicates that when changing M&E systems, an SSP should be prepared to offer extensive training for staff who have disparate levels of comfort and experience with data collection and M&E. An additional strategy to make changing data systems both feasible for staff and aligned with client needs, would be moving toward more efficient data collection systems with actionable analysis to bolster M&E capacity. However, this can be difficult to balance with reporting requirements which are often set by external entities (e.g., local policies or funders) [6, 22].

The primary difference between the high and low M&E SSPs was their access to funding and the M&E resources that accompanied funding (Fig. 1). Notably, all high M&E SSPs had a staff position dedicated to data collection and/or analysis. Most SSPs were under-resourced and without dedicated staff time for M&E, data analyses would fall on staff who have other responsibilities. Our findings are important because they highlight a need for improved financial resources at SSPs specifically dedicated for data collection and M&E as well as reduced reporting requirements that may lessen the data collection burden. Frequently, SSPs reported that most of their data collection was driven by reporting requirements, and they often did not receive feedback on the data they submitted to external entities. This is consistent with findings from a survey of SSPs, where authors found that only ~ 22% of required data reports from SSPs received any feedback [6].

Based on these findings, we have four recommendations to improve M&E capacity at SSPs. First, it is imperative that SSP reporting requirements are minimized and streamlined across funders and governmental stakeholders. This may include more low-barrier reporting requirements such as, not requiring de-duplicated data about SSP participants or allowing narrative reporting rather than quantitative reporting for some SSP outcomes. Second, funders of harm reduction programs should consider harm reduction principles, such as non-judgmental service provision and accepting individuals where they are rather than prioritizing cessation of use, when building reporting requirements. This includes developing data and evaluation plans in partnership with people who use drugs[24]. Thoughtful consideration is needed to determine that the variables collected and the data requested of SSPs align with the services they are providing, ensuring that data collection is internally meaningful. People who use drugs are a highly criminalized and stigmatized population, thus collecting certain types of information about SSP participants (some potentially identifying) is often counter to harm reduction principles.Third, SSPs need increased access to funding to hire staff with the technical skills and bandwidth to focus on data management and analysis. Funding opportunities specifically tied to building data systems or evaluation of existing data are important for these activities to occur. Furthermore, there needs to be funding for data collection initiation and infrastructure for SSPs that lack a system and/or baseline data that would allow them to apply for funds. In our interviews, we saw that it is difficult to overhaul a data system within the context of SSP daily activities and specific funding may make this activity more feasible. Finally, we recommend that funders provide reports and/or analyzed data back to SSPs. SSPs that have low M&E capacity could use these data to monitor trends and changes within their programs, as well as use these data to apply for additional funding.

One of the strengths of our study was the geographic spread of the SSPs interviewed which reflected a variety of policy environments in which SSPs were operating. In addition, study SSPs represented a variety of service delivery models, organizational structures, budgets, and funding systems. Using the CFIR for our data collection and analysis provided the opportunity to compare with other harm reduction studies that used the same framework, and understand commonalities and differences in opportunities and challenges at SSPs. Limitations of our study included our small sample size. By using the NASEN directory to sample SSPs, we were also limited to SSPs that had opted into being featured in the directory, which may not be representative of all SSPs. Because of this – along with the profound diversity in the contexts in which SSPs operate – the generalizability of our study may be limited. Despite the small sample size, however, we did see thematic saturation in our interviews. Due to the exploratory qualitative nature of this study, we did not collect and compare quantitative metrics (e.g., program budget), which may have demonstrated more structural differences between high and low M&E SSPs. Lastly, the CFIR constructs may not encompass all relevant areas of interest for this topic and setting. In anticipation of this, we took both an inductive and deductive approach when designing our codebook to allow for flexibility to add codes derived from the interviews.

## Conclusions

While data collection at SSPs is almost ubiquitous, there is little information about facilitators and barriers to data collection and use at SSPs. In our study, we found that data systems at SSPs vary and are primarily designed to respond to participant needs, but external funding influences the build and alterations to SSP data systems and strong data systems often facilitate further access to funding. Our findings highlight a need for conversations between SSPs and funders about reporting requirements and an opportunity for funders to provide further support to SSPs by offering data analysis and feedback. Reports from funders may provide low M&E SSPs with data summaries that are outside their current analytic capacity and could be utilized for further funding. In addition, we encourage funders to be more transparent with their aggregate findings to increase the value and impact of these data with SSPs. Finally, harmonization is needed in the quantitative indicators collected across funders, while minimizing data collection to the most essential data points needed to effectively evaluate services and monitor trends. It is integral that SSPs are able to maintain focus on providing low-barrier client services, and not collecting data to meet reporting requirements.

### Electronic supplementary material

Below is the link to the electronic supplementary material.

Supplementary Material 1

## Data Availability

The data generated and analyzed during this study are available from the corresponding author on reasonable request. A revised version of the interview guide used during data collection is available as a supplemental material.

## Abbreviations

CFIR: Consolidated Framework for Implementation Research
M&E: Monitoring and evaluation
SSP: Syringe services programs
