# Supplementary material for: Facilitators and barriers to monitoring and evaluation at syringe service programs
Source: Harm Reduct J. 2024 Aug 28;21:157. doi: 10.1186/s12954-024-01073-z (PMC11351253; doi:10.1186/s12954-024-01073-z)
Supplement: Supplementary file 1 — Supplementary Material 1 [file 12954_2024_1073_MOESM1_ESM.docx]

**Supplemental material I: REVISED Interview Guide**

**We’d like to start by learning a bit more about your organization generally, before we start discussing data monitoring specifically.**

**What services do you provide at your organization?**

**Can you describe the personnel structure of your organization? How many people work or volunteer there, and what are some of their different roles?**

Is there a position at your organization that is specifically in charge of collecting and/or analyzing data?

**What are some examples of your organization’s goals and priorities at the moment?**

- - If not addressed: Do you have SSP-specific goals?
  - How (and when) did you come up with these goals? (*Construct: Organizational Incentives & Rewards)*
  - How do you know if your SSP services are successful? What evidence do you use to determine whether or not things are going well?
  - How have these goals changed over time? Are they new or longstanding goals?
  - Do you have a long-term vision for growth? If so, please describe.

**Where does the majority of your organization’s funding come from? (*Construct: Available resources)***

What are advantages to receiving funds from this source?

What are challenges to receiving funds from this source?

Do you have any reporting requirements associated with any of your funding sources? If yes, could you tell us about them and how you meet/met them?

1. **Now, I’d like to ask you some questions specific to “data collection” at your program. Data includes information that is routinely tracked such as demographic information about clients, the number and types of services provided to a client, the number of client encounters that occurred over a day or even non-numerical information such as requests from clients for particular supplies/services or client stories. Does your organization currently collect any data?**

- If yes, what type of data does your program collect? (If no, the next question addresses organizational culture related to data collection)
- How did you decide to collect these variables?
- How do you track these variables (for instance, on pen and paper or do you use a computer system?)
- Is there any data that you wished you track, that you currently don’t, and why?
- How is the data you collect used?

1. **Could you please describe your organization’s culture in regard to data collection? And, by organizational culture, I mean the general beliefs and values of people within your organization. (*Construct: Organizational culture)***

- How does this impact the type and amount of data your program collects?
- What are the beliefs about collecting data that inform this culture?
- What are the legal or cultural barriers where you are that impact what data you collect?

1. **How do people within the organization feel about the amount of data it collects, its process for collecting data, and how it evaluates that data?**

- Do people in the organization feel like there is a strong need for a data system overhaul/updating in your organization? (*Construct: Tension for change*)^2^ Why or why not?
- If not addressed in the response: To what extent do current data collection/monitoring activities fail to meet existing needs?
- If not addressed in the response: How would people at your organization feel about changing the systems you use and/or collecting more data? (*Construct: Implementation Climate*) How would you propose starting that process or conversation at your organization?

1. **What would make members of your organization feel comfortable about data systems? (*Construct: Evidence Strength & Quality*)**

- What type of information would staff need? (i.e., need to know that it would benefit the SSP, that it would lead to more funding, that there would be funding to support data collection/utilization, etc.)
- What type of evidence you would like to share with clients? The community?

1. **What would be some advantages and disadvantages of changing how you collect data?**

- *If not addressed in the response:* How would changes to your data system/collection impact the way you provide services to clients? Would it introduce any new barriers and/or facilitators to clients accessing services?
- Do you believe that updating/launching a new data system could help you meet any of your organizational goals?

1. **How challenging is it to introduce changes or new systems during SSP activities? *(Construct: Complexity)***

- Who are the most influential individuals to engage when deciding to make changes at your SSP? What is the best way to engage them? (Construct: *Engaging: Opinion leaders*)
- Do you need to communicate with clients about changes made at the SSP? If so, who usually communicates these changes and how does it happen? In general, how involved are clients in making decisions about organization changes?
- Would it be similarly challenging to introduce changes related to data systems during SSP activities?
- If you were deciding to change your data system, would you want to engage your clients about how they would feel about the exchange collecting more and/or different data? If so, how would you go about engaging your clients?

1. **Now, thinking about data collection/systems specifically, how would a data system ideally need to be adapted to fit your organization’s needs?**

- What would be the best way to implement a data systems change at your organization?
- What kind of changes to your organization would be needed to accommodate a data collection change?
- What kind of trainings would need to take place to launch/maintain a data systems related change? (i.e. technical trainings, trainings on data collection basics, basic computer trainings, trainings on how to use data to inform decision making?)

1. **If you were to update or implement a new data system at your organization, would you expect to have sufficient resources available to launch and administer an updated system? (*Construct: Available resources)***
   - *If not addressed in answer:*  What resources are you counting on? What resources would you still need?
   - How confident are you that you would be able to effectively manage that data system (*Construct: Self-efficacy)*
   - Do you think your colleagues generally feel confident that they could implement a newly updated/launched data system at your organization?
   - What gives them that level of confidence (or lack of confidence)?
2. **If a new data system was going to be launched, what would be your advice for the best way to launch the platform at SSPs generally, and in your organization specifically?**

What are other key aspects of data collection would need to be considered during the development of a new data monitoring system for SSPs?

1. **Is there anything else that you think it’s important for me to know about data monitoring at SSPs generally?**
